# PLCD3 promotes malignant cell behaviors in esophageal squamous cell carcinoma via the PI3K/AKT/P21 signaling

**DOI:** 10.1186/s12885-023-11409-w

**Published:** 2023-09-29

**Authors:** Mengmeng Wang, Mingjun Gao, Yong Chen, Jun Wu, Xiaolin Wang, Yusheng Shu

**Affiliations:** 1 Dalian Medical University, Dalian, 116000 China; 2 Clinical Medical College, Yangzhou University, Yangzhou, 225000 China; 3 Department of Thoracic Surgery, Northern Jiangsu People’s Hospital, No. 98 Nantong West Road, Yangzhou, 225000 China

**Keywords:** PLCD3, Proliferation, Migration, Invasion, Apoptosis, PI3K/AKT

## Abstract

**Background:**

Phospholipase C Delta 3 (PLCD3) is a member of phospholipase C(PLC) Protein and PLCD3 protein plays a prominent role in many cancers. However, little is known about the role of PLCD3 in esophageal squamous cell carcinoma (ESCC).

**Material and Methods:**

We analyzed PLCD3 mRNA and protein expression in ESCC tissues and cell lines by immunohistochemistry, quantitative real-time PCR, and western blot. The correlation between PLCD3 expression and clinicopathological characteristics was also analyzed. CCK8, colony formation, wound-healing, and transwell assays were conducted to measure cell functional alternations. Flow cytometry was performed to assess the apoptosis rate and cell cycle caused by PLCD3 knockdown. Xenograft models in nude mice to clarify the role of PLCD3 in ESCC. Key proteins in the PI3K / AKT signaling pathway after treatment of ECA109 and KYSE150 cells with the AKT inhibitor MK2206 were analyzed by western blot.

**Results:**

PLCD3 was highly expressed in ESCC tissues and cell lines. PLCD3 expression levels correlated with pathologic stage and lymphatic metastasis. PLCD3 knockdown inhibited cell proliferation, migration, invasion, promoted apoptosis, and caused the cell cycle arrest in the G1 phase. PLCD3 overexpression promoted cell proliferation, migration, and invasion. In vivo experiments with xenografts demonstrated that PLCD3 promoted ESCC tumorigenesis. Finally, Overexpression of PLCD3 activated the PI3K / AKT / P21 signaling.

**Conclusion:**

PLCD3 promotes malignant cell behaviors in esophageal squamous cell carcinoma via the PI3K/AKT/P21 signaling and could serve as a potential target for ESCC treatment.

**Supplementary Information:**

The online version contains supplementary material available at 10.1186/s12885-023-11409-w.

## Introduction

There are two major subtypes of esophageal cancer, esophageal squamous cell carcinoma (ESCC) and esophageal adenocarcinoma (EAC). More than 90% of all esophageal cancers in China are ESCC [1]. Because of the lack of clear symptoms and sensitive screening methods at an early stage, most ESCC patients are at an advanced stage of diagnosis. Moreover, the 5-year overall survival (OS) rate of ESCC is only 15 – 25% due to recurrence and metastasis [2]. Currently, the critical role of molecular mechanisms in cancer has received consistent attention, with molecular markers found progressively more involved in cancer diagnosis, risk stratification, and treatment selection. Although several typical molecular markers of ESCC have been discovered, there are many unknown molecules worth exploration and investigation.

Recently, the dysregulation of lipid metabolism during the development of ESCC has attracted increasing attention. Key enzymes and certain lipid components in lipid metabolism are abnormally expressed and dysregulated in ESCC, which can influence tumor progression, including disorders of glycerophospholipid metabolism [3–6]. PLC is a membrane-associated signaling protease involved in constructing biological membranes, storing energy, signaling, and other essential life activities. PLC is activated by a variety of extracellular ligands to hydrolyze phosphatidylinositol 4,5-diphosphate to generate the second messenger inositol-1,4,5-triphosphate (IP3) and diacylglycerol (DAG), leading causes phosphorylation of target proteins by increasing intracellular Ca^2+^ levels and activating protein kinase C (PKC) to achieve the desired biological effect [7]. PLCD3 is a critical metabolic enzyme in the 13 mammalian PLC subtypes, whose function in tumors is not frequently mentioned. It has been reported to be an oncogene in nasopharyngeal and thyroid cancer [8, 9] and a prognostic marker for early-stage pancreatic ductal carcinoma [10]. However, its role in ESCC has not yet been found. Therefore, the study of PLCD3 affecting the biological behavior of ESCC cells can provide a theoretical basis for treatment.

In this study, PLCD3 was significantly upregulated in ESCC tissues and cells. Further studies focused on its biological function in ESCC cells and explored the underlying mechanism of PLCD3 in ESCC carcinogenesis. The results suggest that PLCD3 promotes ESCC proliferation, migration and invasion via the PI3K/ AKT / P21 signaling pathway.

## Materials and methods

### Esophageal cancer specimens and cell lines

An initial collection of tumor and adjacent non-tumor tissues was performed on.

40 patients undergoing ESCC radical resection at the Department of Thoracic Surgery, Northern Jiangsu People’s Hospital (Yangzhou, China) between March 2020 and March 2021. Samples from 22 patients were selected for paraffin tissue embedding, which were diagnosed with ESCC by the pathology department. The clinicopathological characteristics of all patients are presented in Table 1. Informed consent has been obtained from all individuals included in this study. This study was approved by the Institutional Review Board at Northern Jiangsu People’s Hospital. HEEpiC, ECA109, KYSE150, KYSE410, and TE1 cell lines were obtained from the China Cell Resource Center (Shanghai, China). The cells were cultured in RPMI 1640 (Solarbio) supplemented with 10% fetal bovine serum (Procell). The cells were incubated in a humidified incubator (Thermo Scientific, China) with 5% CO2 at 37 °C.

**Table 1:** PLCD3 Relationship between expression and ESCC characteristics of clinical parameters

Characteristic	N	High expression(n(%))	Low expression(n(%))	Pearson X^2^	P
All	40	33 (82.5)	7 (27.5)		
Age(year)				0.153	0.55
≤55	21	17 (51.5)	4(57.1)		
>55	19	16 (48.5)	3(42.9)		
Gender				0.175	0.579
Male	29	25 (75.8)	4(57.1)		
Female	11	8 (24.2)	3(42.9)		
Tumor central location				0.955	0.62
Distal	6	4(12.1)	2 (28.6)		
Mid	24	20 (60.6)	4 (57.1)		
Proximal	10	9 (27.3)	1 (14.3)		
Histological type				3.238	0.198
Ulcerative type	15	13(39.4)	2(28.6)		
Medullary type	13	10(30.3)	3(42.9)		
Other types	12	10(30.3)	2(28.6)		
Pathologic stage				0.929	0.03^*^
Stage I + II	22	20(60.6)	2(28.6)		
Stage III + IV	18	13(39.4)	5(71.4)		
Lymphatic metastasis				8.505	0.011^*^
No	27	21(63.6)	2(28.6)		
Yes	13	12(36.4)	5(71.4)		

* represents *p* < 0.05

### Immunohistochemical staining and scoring

Sliced 4 μm thick paraffin-embedded tissue was dewaxed and hydrated, then boiled in PH6.0 sodium citrate antigen repair solution for 20 min for antigen repair. Peroxidase activity was blocked during incubation in endogenous peroxise-blocking solution for 15 min. Sections were incubated with anti-PLCD3 (abmart, code:PK83857S, 1: 50 dilution) overnight. After being placed at room temperature and combined with the secondary antibody, IgG antibody (Servicebio, product number: G1215-200T), DAB was used as a chromogen, and hematoxylin was stained with the cell nucleus.

The evaluation of the positive rate of PLCD3 in tissues, The details are as follows: staining intensity score 0 (negative), 1 (weak), 2 (moderate), 3 (strong), and positive cell ratio score 0 (0–5%), 1 (6–25%), 2 (26–50%), 3 (51–71%), 4 (over 75%), The score reference plot is shown in Supplementary Materials (Supplementary material: Figure S1), with the final score assigned as the product of staining intensity and positive rate cell scores.

### RNA extraction and quantitative real-time PCR (qRT-PCR) assay

The TRIzol reagent (Vazyme) was used to extract RNA from tissues and cells. The cDNA was synthesized using the Hifair^®^ III 1st Strand cDNA Synthesis SuperMix for qPCR (gDNA digester plus) (Yeasen Biotechnology, Shanghai, China). Hieff^®^qPCR SYBR Green Master Mix (High Rox Plus) (Yeasen Biotechnology, Shanghai, China) was used to perform the quantitative real time PCR in the StepOne Plus Real-Time PCR System (Applied Biosystems). The relative expression levels of PLCD3 mRNA were normalized to GAPDH as endogenous control respectively by using the 2^−ΔΔCt^ method. The primer sequences are presented in Table 2.

**Table 2:** Primers Sequencing for qPCR.

Primers	Sequences(5ʹ-3ʹ)
PLCD3-Forward	CCACAACACCTATCTGACTGAC
PLCD3-Reverse	CTGGGCAAAGGCCCTAACAT
GAPDH-Forward	TCATTTCCTGGTATGACAACGA
GAPDH-Reverse	GTCTTACTCCTTGGAGGCC
PLCD3-siNC-sense	UUCUCCGAACGUGUCACGUTT
PLCD3-siNC-antisense	ACGUGACACGUUCGGAGAATT
PLCD3-siRNA1-sense	GCAGCUCAUUCAGACCUAUTT
PLCD3-siRNA1antisense	AUAGGUCUGAAUGAGCUGCTT
PLCD3-siRNA2-sense	GCCCACUACUUCAUCUCUUTT
PLCD3-siRNA2-antisense	AAGAGAUGAAGUAGUGGGCTT
PLCD3-siRNA3-sense	GCCACGCUCUUCAUCCAAATT
PLCD3-siRNA3-antisense	UUUGGAUGAAGAGCGUGGCTT

### Western blot (WB) assay

The whole cell or tissue mixture was isolated using RIPA lysate (Solarbio, Cat: R0020), PMSF, protease inhibitor, and protein phosphatase inhibitor mixture, and equal amounts of protein were separated on 10%SDS-PAGE and transferred to a PVDF membrane (Immobilon-P, cat:IPVH00010).They were blocked using 5% skim milk and incubated overnight with primary antibody PLCD3 (Abmart, code:PK83857S), anti-GAPDH (Proteintech, cat:10494-1-AP), MMP2 (Cell Sinaling, cat:40,994), MMP9 (Cell Sinaling, cat:15,749), p-PI3K (Cell Sinaling, cat:17,366), t-PI3K (Cell Sinaling, cat:9655), p-AKT (Cell Sinaling, cat:13,038 S), t-AKT (Cell Sinaling, cat:4685 S), P21 (Cell Sinaling, cat:2947), Bax (Cell Sinaling, cat:5023T), Caspase3 (Cell Sinaling, cat:9664T) and Bcl2 (Cell Sinaling, cat:4223T) binding 4 °C. Then they were incubated in secondary antibody IgG (ABclonal, lot:9,300,014,001) for 2 h at room temperature. Protein blots were cut prior to hybridisation with antibodies during blotting. Protein bands were visualized using Super ECL Detection Reagent(Yeasen Biotechnology, Shanghai). Image J software performed the protein band gray-scale analysis.

### RNA oligo and plasmid transfection

The siRNA and BamHI RcoRI pcDNA3.1 was purchased from GenePharma (Shanghai, China). The siRNA sequences are presented in Table 1. The cells were incubated in 6-well plates, and transfection started when cell density reached 50%. GP-transfect-Mate (GenePharma) was used to perform the transfection. The transfection efficiency was detected by qRT-PCR and WB methods.

### CCK-8 assay

The proliferation assay were performed on a panel of 96 wells with 1 × 10^3^ cells per well. After 24, 48, 72, and 96 h, 10µL of CCK-8 solution (Yeasen) was added to every well and then incubated for 1 h 30 min. The absorbance (OD) of each well at 450 nm was detected by an enzyme labeling instrument(Skanlt RE 7.0).

### Colony formation assay

Cells after siRNA or BamHI RcoRI pcDNA3.1 transfection were seeded at a density of 1 × 10^3^ cells per well in 6 or 12-well plates, during which fresh medium containing 10%FBS was replaced on time. Two weeks later, the cells were fixed with 4% paraformaldehyde for 15 min, stained with 0.1% crystal violet solution for 12 min, air-dried and photographed, and colony counts were performed with Image J software.

### Wound healing assay

Wound-healing assays were performed to assess the migration ability of the cells. Transfected cells (15 × 10^4^/well) were seeded in six-well plates and incubated continuously until the monolayer of cells were evenly distributed at the bottom of the plate. A micropipette gun head uniformly and slowly forms a scratch wound on the surface of the cell. The cells were washed with PBS several times to remove all floating cells. Then residual cells were cultured with RPMI 1640 without FBS. Wounds were then taken at 0, 24, 48 h under an inverted microscope (OLYMPUS-CKX53, China). Images were analyzed using the Image J software.

### Transwell assay

Transwell assay was performed to assess the migration and invasion ability of the cells. Cells were transfected in 6-well plates and incubated after 48 h before starting the experiments. Cells were washed twice with PBS, cells were digested with 0.25% trypsin cell digestive solution (Beyotime) and centrifuged, and cells were resuspended in serum-free 1640 and counted. Then 200ul of cell suspension was added to the upper layer covered with matrix (BD Biocoat) or no matrix free chamber (Corning), while 500ul of 1640 containing 10%FBS was added to the lower chamber. The transwell chamber was grown in a cell culture incubator for 48 h. The cells were then fixed with 4% paraformaldehyde for 15 min, stained with 0.1% crystal violet solution for 7 min, then washed twice with PBS, and the cells on the upper surface of the bottom chamber were gently wiped away with a cotton swab. Cells on the lower surface of the bottom of the chamber were left dry, and images were taken with an inverted microscope (OLYMPUS-CKX53). Images were analyzed using the Image J software.

### Flow cytometry analysis

Cell cycle and apoptosis were measured using flow cytometry. For the cell cycle, the cells were washed twice with PBS and digested with trypsin, 70% ethanol for 4 h at 24**°**C, it was subsequently incubated in 500 µl of configured propidium iodide staining solution (PI) (Beyotime) at 37**°**C for 30 min. With regard to cell apoptosis, cells were washed twice with PBS and digested with trypsin, add the Annexin-FITC detection reagent (Beyotime) in turn.Then the cells were detected using a FACS flow cytometer (BD Biosciences, CA, USA), and analyzed the results using the Flow Jo software.

### Functional enrichment analysis

To further investigate the function between PLCD3 and the upregulated genes caused by it, KEGG (www.kegg.jp/kegg/kegg1.html) and GO enrichment analysis was performed by clusterProfiler (version 3.14.3) R software [11–13]. Among them, GO enrichment includes biological processes (BP), molecular functions (MF) and cellular components (CC). The minimum gene set was set to 5, the maximum gene set to 5000, P value of < 0.05, and an FDR of < 0.25 were considered statistically significant. PLCD3 was divided into two groups of samples with high and low expression, and the correlation between PLCD3 expression and PI3K/AKT pathway genes was analyzed using GSEA software.

### Xenograft model

The animal experiments were performed with the approval of the Experimental Animal Ethics Committee of Yangzhou University. A mouse xenograft model was established to explore the functional role of PLCD3 in vivo. The ECA109 cells were spread in six-well plates and then transfected with siNC, siRNA2 and siRNA3. 15 Balb / c nude mice (Nanjing JiBiological Co., LTD) were randomly divided into 3 groups to inject the above treated cells (6 × 10^5^) in the axilla of nude mice. Tumor volume was monitored every 5 days, the formula was: V = (Length x Width^2^) x 0.5, nude mice were killed 5 weeks later, and tumor weights were recorded. Anestheia using isoflurane and subsequently nude mice were killed by cervical dislocation. Animal experiments were performed in accordance with the animal care guidelines and were approved by the ethics committee.

### Statistical analysis

All experimental data were treated with GraphPad Prism8.0 and displayed with the mean value plus or minus the standard deviation. Student’s test was used to compare differences between data from the two groups, and Dunnett’s test in Ordinary one-way ANOVA to compare differences between multiple groups. *P* < 0.05 were considered statistically significant.

## Results

### PLCD3 expression is distinctly elevated in ESCC tissues and cells

**Fig. 1:**
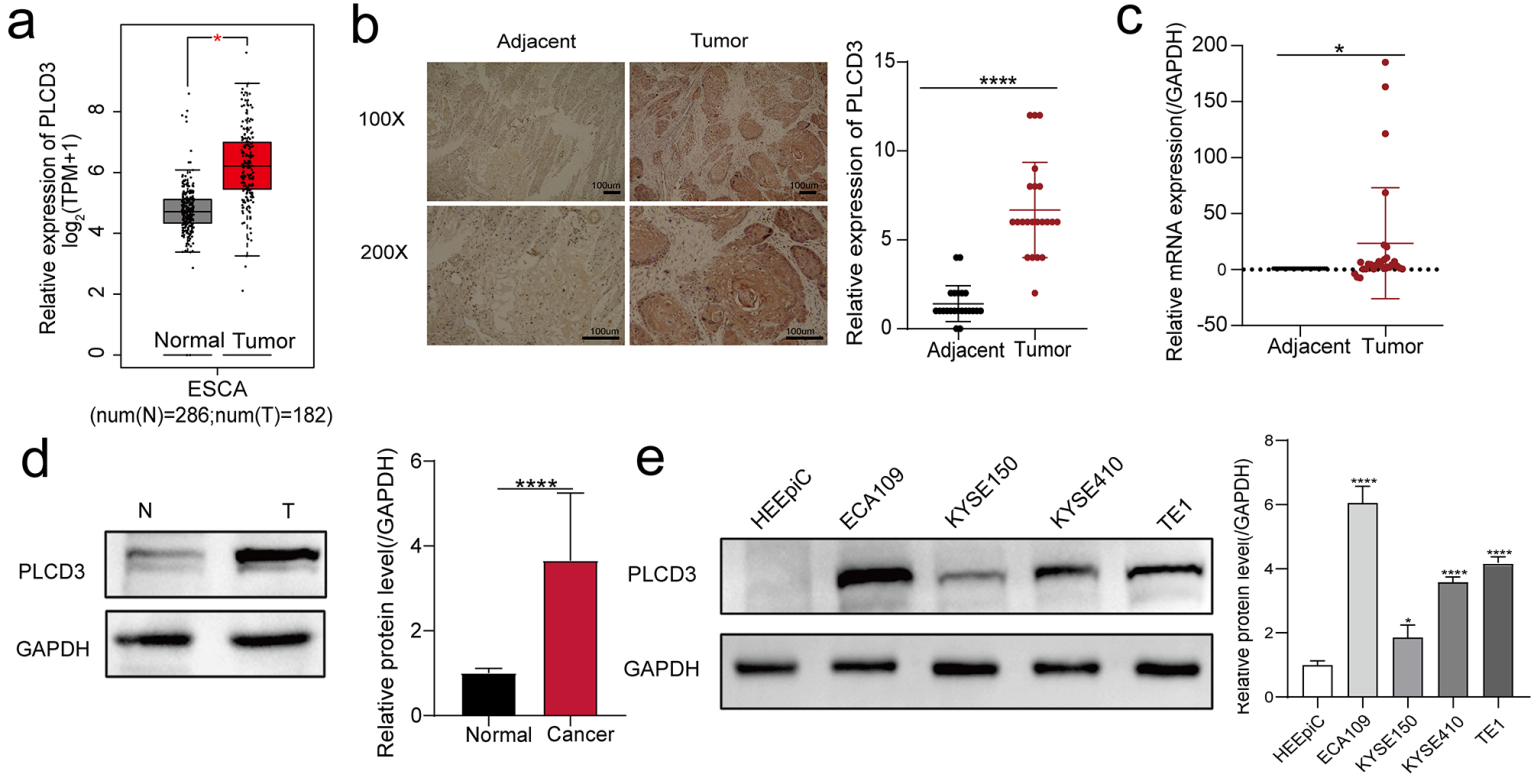
PLCD3 expression was upregulated in ESCC tissues and cells (**a**) We searched the GEPIA website for the expression profile of PLCD3 in 182 esophageal cancer tissues compared with 286 normal tissues. (**b**) PLCD3 expression was detected in 22 paired ESCC tissues and adjacent non-tumor tissues by IHC method. (**c**) PLCD3 expression was detected in 10 pairs of ESCC tissues and adjacent non-tumor tissues by qRT-PCR. (**d**) PLCD3 expression in 20 paired ESCC tissues, and adjacent non-cancerous tissues was measured by WB (only 1 pairs is in the text and all bands are shown in Supplementary material). (**e**) The expression of PLCD3 in ESCC cells (ECA109, KYSE150, KYSE410, TE1) and HEEpiC was determined by WB. *p < 0.05, ****p < 0.0001

Based on the GEPIA (http://gepia2.cancer-pku.cn/# index), the boxplot showed that PLCD3 was significantly upregulated in 182 esophageal cancer tissues compared with 286 normal tissues (Fig. 1a ). Then, IHC, qRT-PCR, and WB experiments were used to verify the RNA and protein levels of PLCD3 in ESCC and normal tissues, and the results indicated that the expression of PLCD3 was significantly increased in ESCC tissues (Fig. 1b-d). Further analysis of the differential expression in ESCC cell lines and the normal esophageal epithelial cell line (HEEpiC) suggested that the expression of PLCD3 was higher than that of HEEpiC (Fig. 1e).

To investigate the clinical significance of PLCD3 upregulation in ESCC, the relationship between the clinicopathological features of ESCC cases and PLCD3 expression levels was further analyzed. We found significant differences between high PLCD3 expression in Pathologic stage and Lymphatic metastasis (*p* = 0.03, *p* = 0.01, respectively, Table 1). That is, high stage PLCD3 expression was significantly higher than low stage, and PLCD3 expression in the lymphatic metastasis group was higher than non-lymphatic metastasis.

### PLCD3 knockdown inhibits tumor cell proliferation, migration, and invasion

**Fig. 2:**
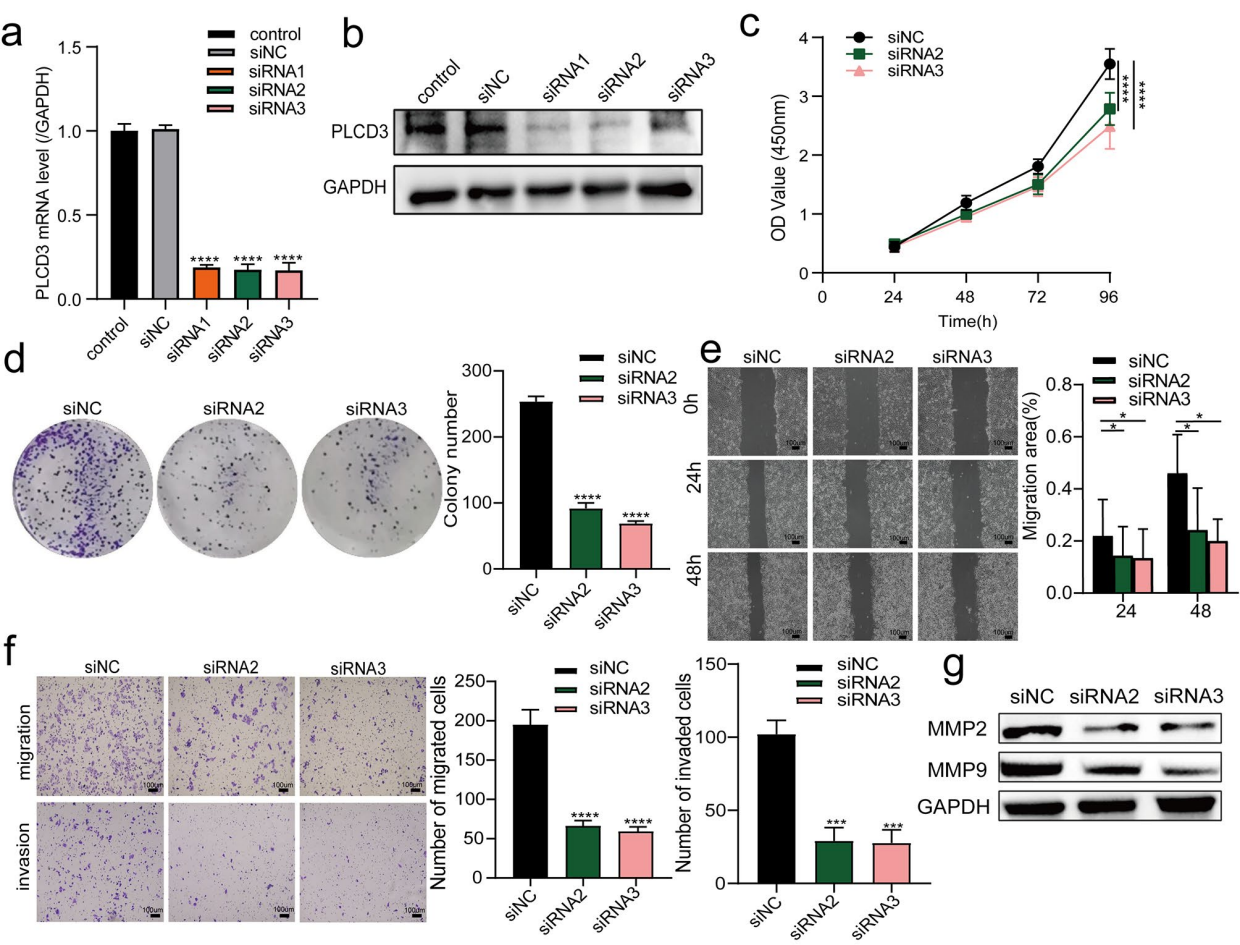
PLCD3 knockdown inhibited tumor cell proliferation, migration, and invasion (**a**, **b**) The efficiency of PLCD3 knockdown in ECA109 cells transfected with siRNA1 / 2 / 3 was determined by both qRT-PCR and WB. control: no siRNA infection; siNC: negative control. (**c**, **d**) The viability and proliferation ability of ECA109 cells transfected with siNC, siRNA2 and siRNA3 were determined by CCK8 and clone formation assay. (**e**) Wound healing assay examining the mobility of ECA109 cells transfected with siNC, siRNA2 and siRNA3. (**f**) The number of ECA109 cells transfected with siNC, siRNA2 and siRNA3 migrated and invaded were assessed by transwell assay. (**g**) WB measured the expression levels of migration-related proteins (MMP2, MMP9) in ECA109 cells transfected with siNC, siRNA2 and siRNA3. *p < 0.05, ***p < 0.001, ****p < 0.0001

To determine the effect of PLCD3 knockdown on ESCC cell growth, the downregulation of endogenous PLCD3 levels in highly expressing ECA109 cells using RNAoligo-mediated siRNA and verified the ECA109 cell knockdown efficiency by qPCR and WB test results (Fig. 2a,b), The best two siRNA were then selected for a series of functional assays. PLCD3 depletion significantly inhibited the cell viability of ECA109 cells (Fig. 2c,d). Wound healing and transwell assays showed that PLCD3 knockdown led to a significant decrease in cell migration capacity, and its invasion capacity was also sharply reduced (Fig. 2e,f).Then WB examined the tumor invasion-related molecules MMP2 and MMP9,and suggested that PLCD3 knockdown caused its diminished molecular expression (Fig. 2g).

### PLCD3 overexpression promotes tumor cell proliferation, migration, and invasion

**Fig. 3:**
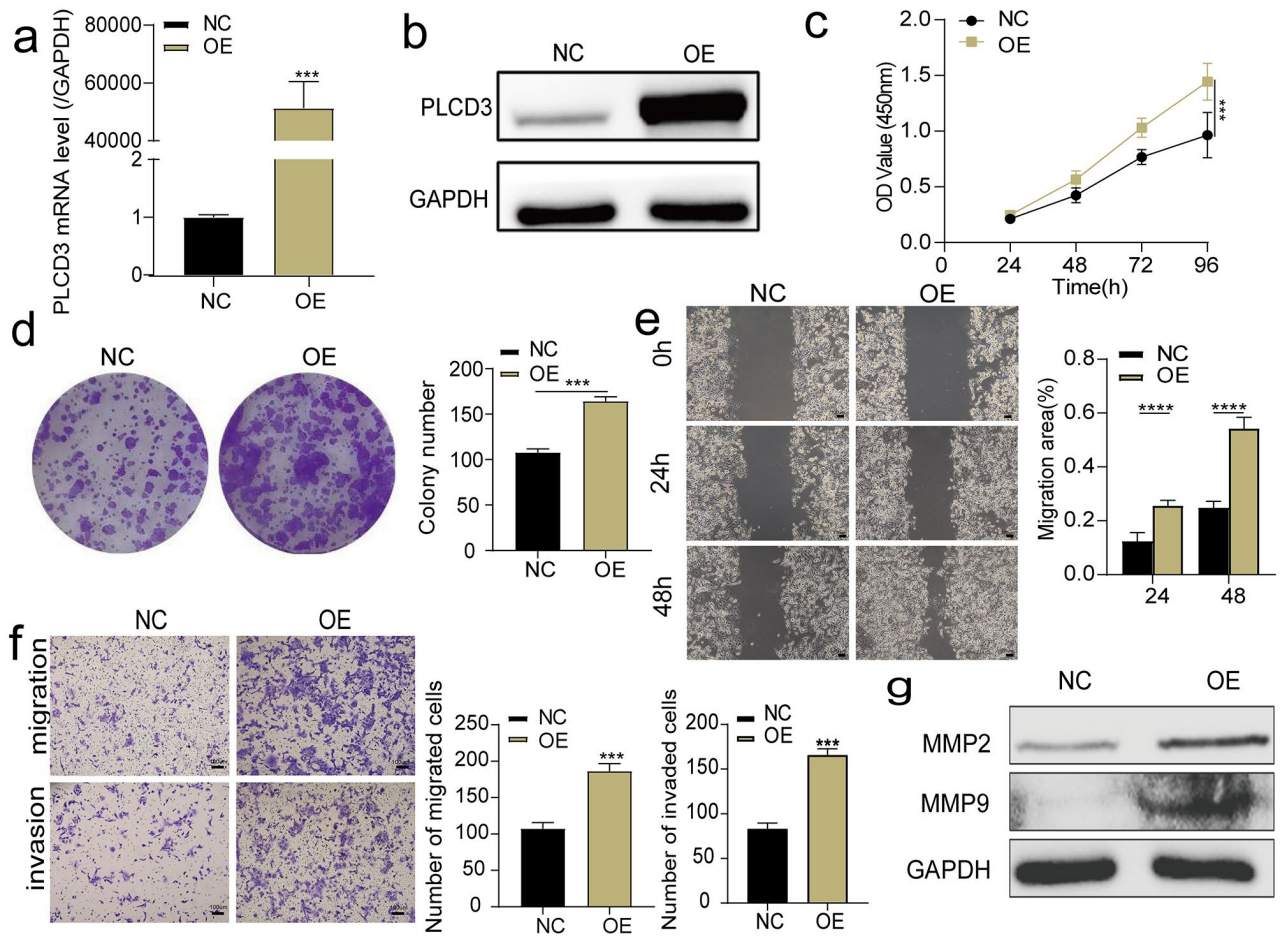
PLCD3 overexpression promoted tumor cell proliferation, migration, and invasion (**a**, **b**) The efficiency of upregulating PLCD3 in KYSE150 cells with transfected plasmid pcDNA3.1 was tested by both qRT-PCR and WB. NC, the cells were treated with the negative control. OE, the cells were treated with PLCD3 overexpression. (**c**, **d**) Viability and proliferation of KYSE150 cells transfected with NC and OE were examined by CCK8 and clone formation assay. (**e**) Wound-healing assay examining the mobility of KYSE150 cells transfected with NC and OE. (**f**) The number of cells migrated and invaded by KYSE150 cells transfected with NC and OE was determined by Transwell assay. (**g**) WB measured the expression levels of the migration-related proteins (MMP2, MMP9) in KYSE150 cells transfected with NC and OE. *p < 0.05, **p < 0.01, ***p < 0.001, ****p < 0.0001

The above experiments found that PLCD3 was expressed at the lowest level in KYSE150 cells, therefore, we used the PLCD3 plasmid to promote the endogenous expression of PLCD3 in KYSE150 cells. After transfection with PLCD3, PLCD3 mRNA and protein levels in KYSE150 cells increased significantly (Fig. 3a,b), and subsequent CCK8 and colony formation experiments showed that PLCD3 upregulation significantly increased the proliferation capacity of cells (Fig. 3c,d). In addition, the results of wound-healing and transwell experiments suggested that the upregulation of PLCD3 led to a significant improvement in cell migration and invasion capacity (Fig. 3e,f). Meanwhile, the expression of invasion-related molecules MMP2 and MMP9 increased significantly (Fig. 3g).

### PLCD3 is involved in the transition from G1 to S phase and apoptosis

**Fig. 4:**
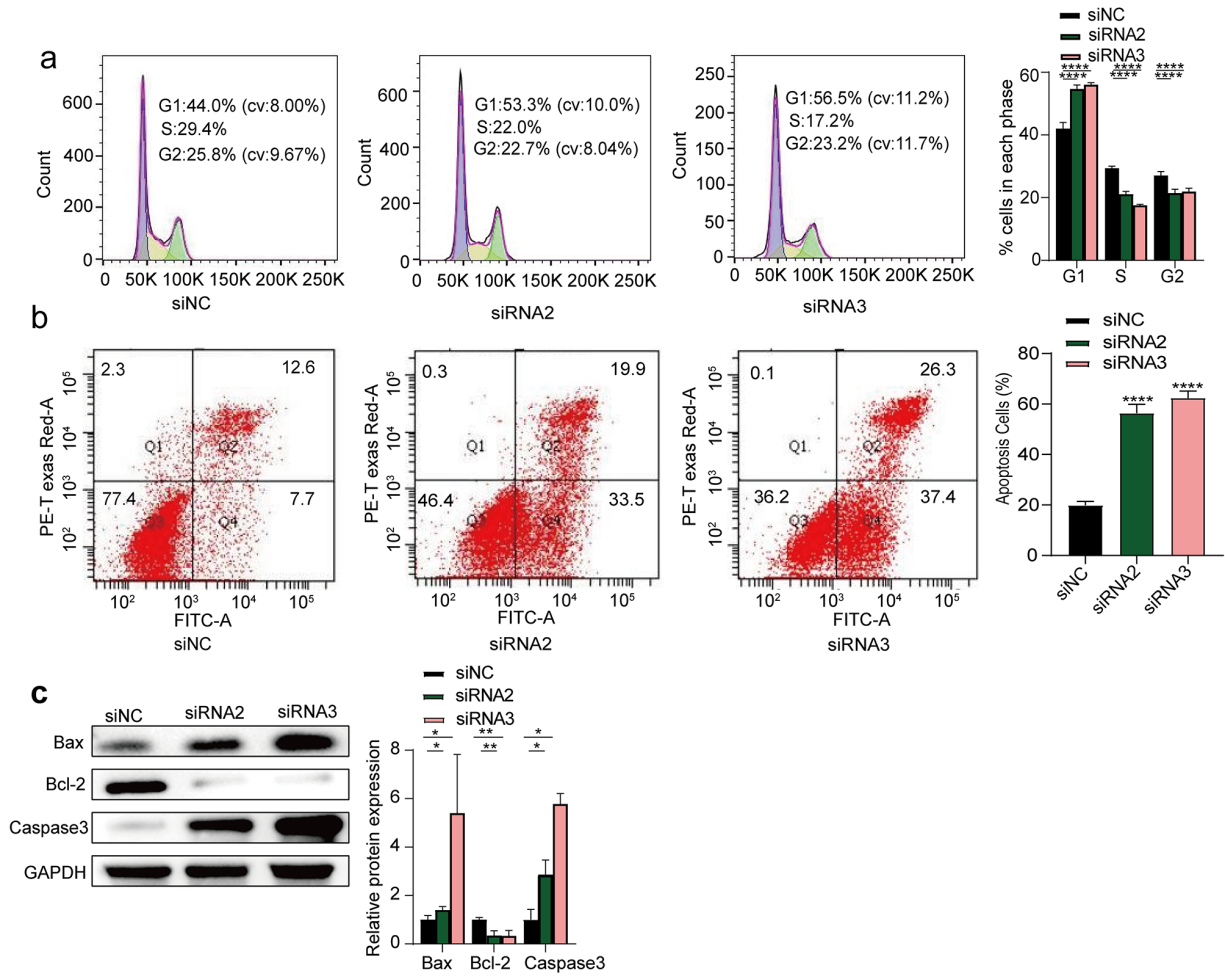
PLCD3 is involved in the transition from G1 to S phase and apoptosis (**a**) The effect of PLCD3 knockdown on the cell cycle of ECA109 cells was determined by Cell cycle and Apoptosis Analysis Kit. (**b**) The effect of PLCD3 knockdown on apoptosis in ECA109 cells was tested by the Annexin V cell apoptosis detection kit. (**c**) The expression levels of apoptosis-related proteins (Bax, Bcl-2, Caspase3) in ECA109 cells were measured by WB. *p < 0.05, **p < 0.01, ****p < 0.0001

The above results indicate that PLCD3 upregulation promotes proliferation, migration and invasion of ESCC cells, and PLCD3 silencing has the opposite biological behavior. So, is this change in biological behavior related to the cell cycle and apoptosis? Flow cytometry was subsequently performed to explore whether PLCD3 knockdown caused cell cycle arrest and increased apoptosis in ESCC cells. The results showed that in ECA109 cells, PLCD3 knockdown induced cell cycle arrest in G1 phase (Fig. 4a) and promote cell apoptosis(Fig. 4b). To further verify these results, the expression of apoptosis-related proteins was analyzed by WB. We found that apoptosis-associated key regulatory proteins were significantly altered in PLCD3 knockdown cells, namely up-regulation of Bax and Caspase3 and downregulation of Bcl-2 (Fig. 4c). In conclusion, PLCD3 is critical for G1 to S phase transition and apoptosis in the ECA109 cell cell cycle.

### PLCD3 exerts its biological effects through the PI3K / AKT / P21 signaling pathway

**Fig. 5:**
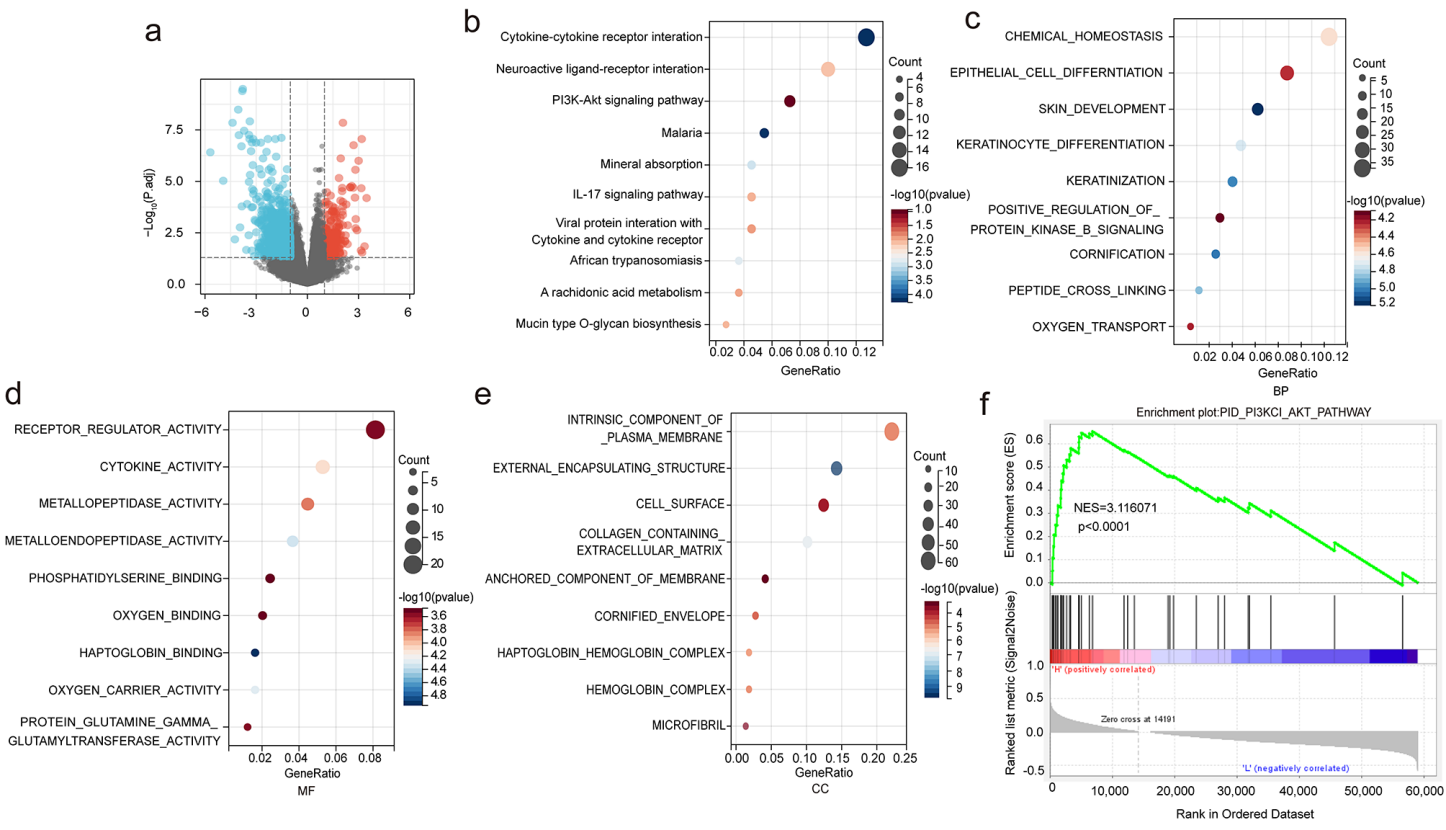
PLCD3 promotes PI3K/AKT pathway activation (**a**) Volcano plot of DEGs (adjusted p < 0.05 and fold change > 2), including 522 upregulated genes. (**b**) KEGG enrichment analysis of upregulated DEGs. (**c**, **d**, **e**) GO enrichment analysis of upregulated DEGs. (**f**) GSEA analysis of the correlation between PCLD3 expression and the PI3K/AKT pathway gene signature

As changes in PLCD3 expression have distinct effects on the ESCC cell phenotype, we next explored the mechanism of PLCD3 action in ESCC. To analyze the changes in gene expression and pathways potentially affected by PLCD3, a differential gene analysis was first performed. We observed that the expression level of PLCD3 can significantly changed hundreds of genes Supplementary Materials (Supplementary material: Table S1) at the RNA level (Fig. 5a). The upregulated genes Supplementary Materials (Supplementary material: Table S2) were subjected to KEGG and GO enrichment analysis. KEGG showed that the upregulated genes were mainly involved in cytokine interaction, PI3K-AKT signaling pathway, mineral absorption and arachidonic acid metabolism (Fig. 5b). Biological processes (BP) indicates that the upregulated genes are involved in processes such as cell differentiation, keratinization, positive regulation of protein kinase B signaling pathway and so on (Fig. 5c). Molecular functions (MF) indicates that upregulated genes involved in receptor regulator, cytokine, metallopeptidase et al. (Fig. 5d). Cellular components (CC) indicates upregulated genes that constitute the plasma membrane of cells, cell surface, cellular proteins and cellular components of microfibril (Fig. 5e). In addition, the mRNA expression level of PLCD3 in ESCC was bounded by the median value and divided into high and low expression groups for GSEA enrichment, which showed that PLCD3 upregulation was able to activate the PI3K/AKT signaling pathway (Fig. 5f).

**Fig. 6:**
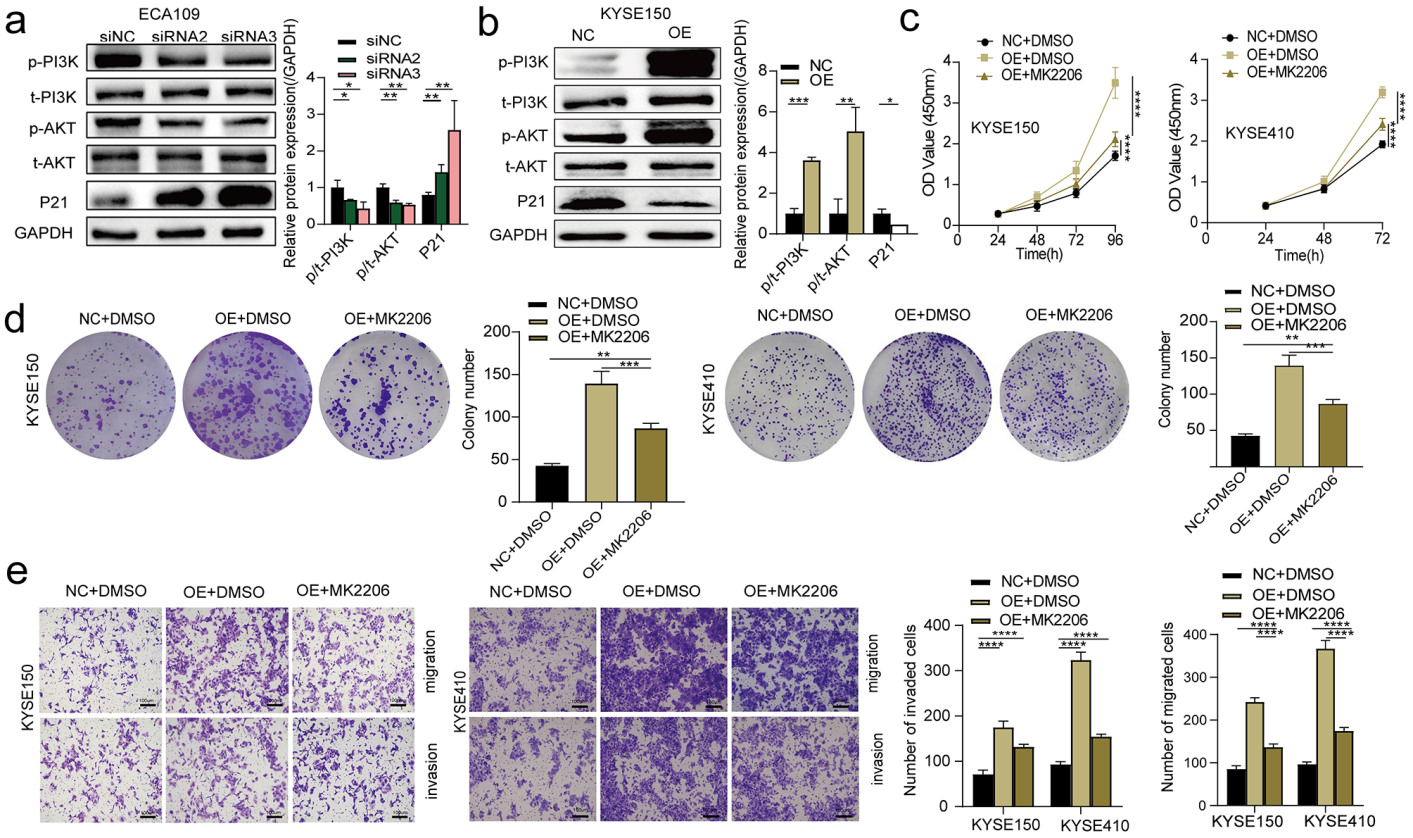
PLCD3 exerts its biological roles through the PI3K / AKT / P21 signaling (**a**) WB was used to detect the levels of p-PI3K, PI3K, p-AKT, AKT, and P21 proteins in ECA109 cells after siNC, siRNA2 and siRNA3 transfection. (**b**) The levels of p-PI3K, PI3K, p-AKT, AKT, and P21 proteins in KYSE150 cells transfected with NC and OE were determined by WB. (**c**, **d**) The viability and proliferation of KYSE150 and KYSE410 cells under different treatments were determined by CCK8 and Clone formation assay: (i) NC + DMSO, (ii) OE + DMSO, (iii) OE + MK2206. (**e**) Transwell detects the migration and invasion ability of KYSE150 and KYSE410 without treatment: (i) NC + DMSO, (ii) OE + DMSO, (iii) OE + MK2206. A 10 mM stock solution was prepared by dissolving MK2206 in DMSO, where the MK22206 experimental concentration was 2 mM and 0.02% DMSO was used as a negative control. *p < 0.05, **p < 0.01, ***p < 0.001, ****p < 0.0001

It is well known that the PI3K/AKT pathway is an intracellular signaling pathway that promotes metabolism, proliferation, cell survival, growth, and angiogenesis, and is an important signaling pathway for the occurrence and progression of ESCC [14]. Therefore, We explored whether PLCD3 could activate the PI3K/AKT pathway to promote tumor development. Protein imprinting results showed that the p / t PI3K and p / t AKT protein levels were decreased, and P21 was significantly increased in ECA109 cells transfected with siRNA (Fig. 6a); p / t PI3K and p / t AKT protein levels increased in pcDNA3.1-transfected KYSE150 cells, and P21 decreased significantly (Fig. 6b). The above results suggest that PLCD3 may promote tumor development by activating the PI3K / AKT / P21 signaling pathway.

To further confirm the involvement of PI3K / AKT / P21 signaling, the AKT inhibitor (MK2206) was used. KYSE150 and KYSE410 cells showed low levels of PLCD3 expression and were treated with PLCD3 overexpression and treated with MK2206. The results suggested that PLCD3 overexpression significantly enhanced cell proliferation, migration and invasion, but MK2206 treatment inhibited the above biological behaviors (Fig. 6c-e). These data suggest that PLCD3 may promote cell proliferation, migration, and invasion through the activation of the PI3K / AKT / P21 pathway.

### PLCD3 promotes tumorigenesis in vivo

**Fig. 7:**
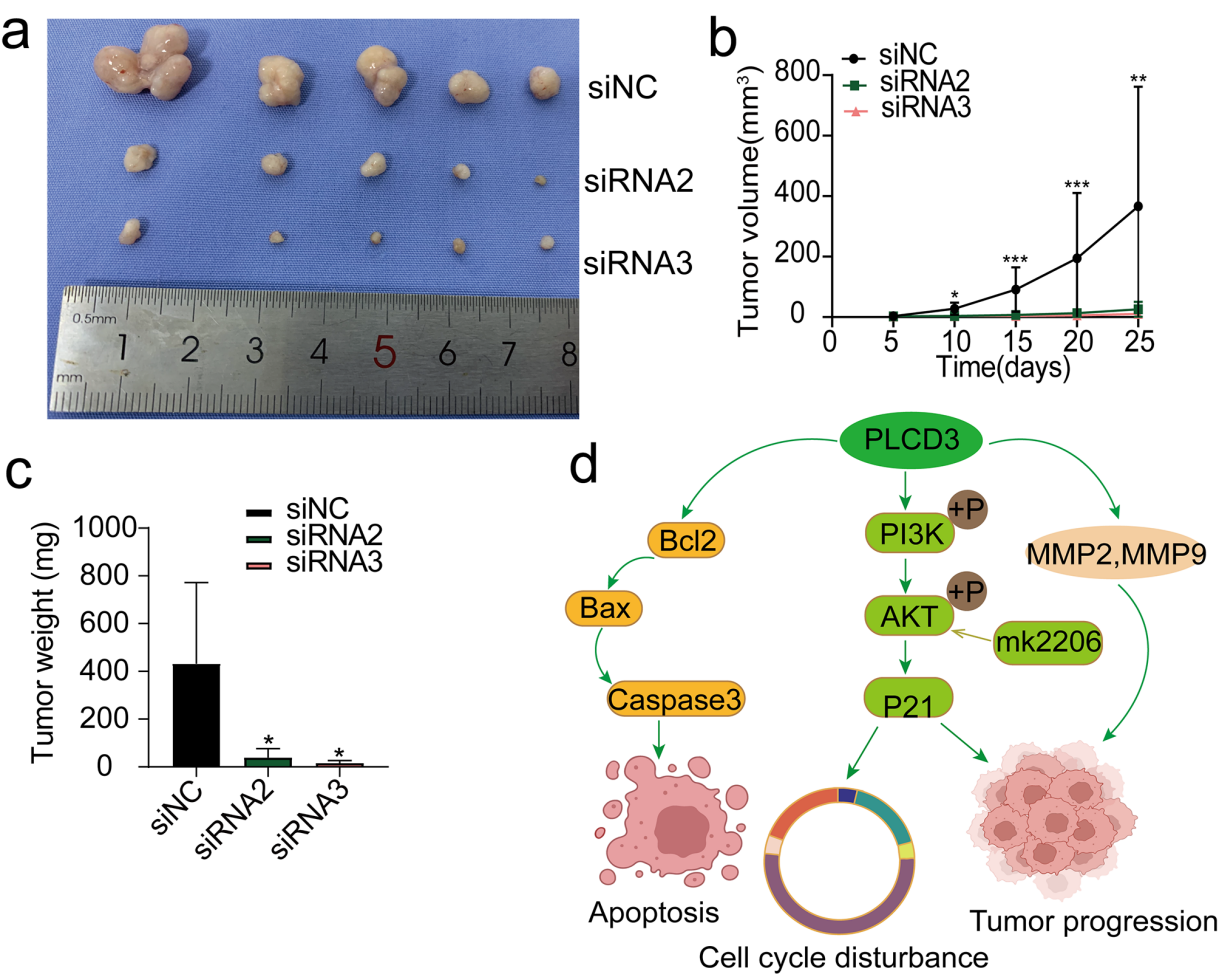
PLCD3 promotes tumorigenesis in vivo (**a**) Xenograft tumors in nude mouse models. (**b**) Tumor volume. (**c**) Tumor weight. (**d**) Schematic representation of PLCD3 promoting the progression of esophageal squamous cell carcinoma. *p < 0.05, **p < 0.01, ***p < 0.001

A xenograft nude mouse model was established to clarify the role of PLCD3 in ESCC in vivo. Tumor changes were closely monitored after injection of ECA109 cells from transfected siNC, siRNA2 and siRNA3. The results showed that PLCD3 knockdown inhibited tumor growth, that is, a significant decrease in tumor volume and weight (Fig. 7a-c). The above results suggest that PLCD3 may promote the malignant biological behavior of ESCC cells by promoting the phosphorylation of PI3K and AKT (Fig. 7d).

## Discussion

As was previously described, PLCD3 plays an oncogenic role in a variety of cancers, such as nasopharyngeal, thyroid, and early pancreatic ductal carcinoma [8–10]. However, whether PLCD3 has the same effect on ESCC progression and how it works is unclear. The current study demonstrated that PLCD3 is significantly upregulated in ESCC tissues and cell lines. PLCD3 promotes the development of ESCC cells by regulating malignant behaviors such as proliferation, migration, and invasion, suggesting the tumor-promoting role of PLCD3 in ESCC.

In previous studies, Lizhi Lin et al. [9] found that the increased expression of PLCD3 was associated with the metastatic stage of thyroid cancer. Nakamura et al. [15] reported that loss of PLCD1 and PLCD3 in mouse embryos increased apoptosis. Our findings are consistent with its report that PLCD3 knockdown inhibited proliferation, migration, invasion and promoted apoptosis of ESCC cells. In vivo experiments show that PLCD3 promoted ESCC tumorigenesis.

Dysregulation of cell migration and apoptosis are two major drivers of cancer progression that are regulated by multiple oncogenic pathways [16–19]. Metastasis is the major cause of cancer-related death, and the migration of and invasion of cancer cells into surrounding tissues and vasculature is an important step in cancer metastasis [20]. Cell migration is a dynamic, complex process that requires multiple interdependent steps, such as cancer cells exhibiting cytoskeletal reorganization, reduced adhesion function, and loss of cell polarity [21]. Migration-induced changes in classical molecular features include increases in mesenchymal markers and changes in matrix metalloproteinases, such as MMP2, and MMP9 [22, 23]. In this study, we found that the expression level of metastasis markers was affected by the changes in PLCD3 expression. Downregulation of PLCD3 decreased the expression of MMP2 and MMP9, while upregulation of PLCD3 showed the opposite results. In addition, we found that the expression levels of Bax and Caspase3 increased while Bcl-2 decreased when PLCD3 knockdown. It is known that apoptosis is one of the key features of cancer [24]. Apoptosis mainly includes the endogenous apoptotic pathway, exogenous apoptotic pathway, and execution pathway, where the key markers of the endogenous apoptotic pathway include Bax and Bcl-2, and Caspase3 proteins belong to the key proteins of the executive pathway [25]. This is fully demonstrated by our study that PLCD3 downregulation caused increased apoptosis.

Furthermore, we sought to investigate the molecular mechanism of PLCD3 as an ESCC oncogene. The PI3K / AKT pathway is frequently over-activated in various tumor types [26]. Several current studies [27–31] showed that the PI3K / AKT pathway can promote ESCC cell growth and metastasis, and the phosphorylation level of both was significantly and positively correlated with the invasion and metastatic ability of cancer cells. This is in line with our finding that PLCD3 downregulation led to a significant reduction in PI3K / AKT phosphorylation, versus PLCD3 upregulation (Fig. 6a,b). P21 is a cyclin-dependent kinase inhibitor involved in cell cycle progression, conducting G1 / S phase arrest and simultaneously inhibiting the proliferation and migration of ESCC cells [32–36]. Our findings suggest that downregulation of PLCD3 caused an increase in P21 expression, and the upregulation of PLCD3 caused a decrease of P21 expression. Meanwhile, PLCD3 downregulation induced a cell cycle GI / S arrest, which was consistent with the elevated P21 expression. Finally, the AKT inhibitor MK2206 significantly impaired the promoting effect of PLCD3 upregulation on cell growth, migration and invasion (Fig. 6c-e). The results suggest that PLCD3 may promote cell proliferation, migration and invasion by activating the PI3K / AKT / P21 pathway.

However, our study still has some limitations. For example, in vivo experiments are needed to further validate the biological function of PLCD3 in ESCC. Meanwhile, PLCD3 acts as a phospholipid metabolism enzyme and its related mechanism of action need to be specifically elucidated.

In conclusion, these results indicate that PLCD3 is upregulated in ESCC, high expression of PLCD3 is closely related to the malignant biological behavior of ESCC, and PLCD3 may play an oncogenic role in ESCC via the PI3K / AKT / P21 pathway. This study may provide potential markers and target genes for the treatment of ESCC.

### Electronic supplementary material

Below is the link to the electronic supplementary material.

Supplementary Material 1

Supplementary Material 2

Supplementary Material 3

Supplementary Material 4

## Data Availability

The datasets used and/or analysed during the current study are available from the corresponding author on reasonable request.

## References

[1] Bennett AN, Huang RX, He Q, Lee NP, Sung WK, Chan KHK (2022). Drug repositioning for esophageal squamous cell carcinoma. Front Genet.

[2] Yang W, Cheng B, Chen P, Sun X, Wen Z, Cheng Y (2022). BTN3A1 promotes tumor progression and radiation resistance in esophageal squamous cell carcinoma by regulating ULK1-mediated autophagy. Cell Death Dis.

[3] Zhu ZJ, Qi Z, Zhang J, Xue WH, Li LF, Shen ZB, Li ZY, Yuan YL, Wang WB, Zhao J (2020). Untargeted metabolomics analysis of esophageal squamous cell Carcinoma discovers dysregulated metabolic pathways and potential diagnostic biomarkers. J Cancer.

[4] Sha Y, Hong H, Cai W, Sun T (2022). Single-cell transcriptomics of endothelial cells in Upper and Lower Human esophageal squamous cell carcinoma. Curr Oncol.

[5] Chang W, Luo Q, Wu X, Nan Y, Zhao P, Zhang L, Luo A, Jiao W, Zhu Q, Fu Y, Liu Z (2022). OTUB2 exerts tumor-suppressive roles via STAT1-mediated CALML3 activation and increased phosphatidylserine synthesis. Cell Rep.

[6] Zhou X, Huang F, Ma G, Wei W, Wu N, Liu Z (2022). Dysregulated ceramides metabolism by fatty acid 2-hydroxylase exposes a metabolic vulnerability to target cancer metastasis. Signal Transduct Target Ther.

[7] Liu Z, Wu X, Wang Q, Li Z, Liu X, Sheng X, Zhu H, Zhang M, Xu J, Feng X, Wu B, Lv X (2022). CD73-Adenosine A(1)R Axis regulates the activation and apoptosis of hepatic stellate cells through the PLC-IP(3)-Ca(2+)/DAG-PKC signaling pathway. Front Pharmacol.

[8] Liu W, Liu X, Wang L, Zhu B, Zhang C, Jia W, Zhu H, Liu X, Zhong M, Xie D, Liu Y, Li S, Shi J, Lin J, Xia X, Jiang X, Ren C (2018). PLCD3, a flotillin2-interacting protein, is involved in proliferation, migration and invasion of nasopharyngeal carcinoma cells. Oncol Rep.

[9] Lin L, Wen J, Lin B, Chen H, Bhandari A, Qi Y, Zheng D, Wang O (2021). Phospholipase C Delta 3 inhibits apoptosis and promotes proliferation, migration, and invasion of thyroid cancer cells via Hippo pathway. Acta Biochim Biophys Sin (Shanghai).

[10] Zhou X, Liao X, Wang X, Huang K, Yang C, Yu T, Han C, Zhu G, Su H, Han Q, Chen Z, Huang J, Gong Y, Ruan G, Ye X, Peng T (2020). Noteworthy prognostic value of phospholipase C delta genes in early stage pancreatic ductal adenocarcinoma patients after pancreaticoduodenectomy and potential molecular mechanisms. Cancer Med.

[11] Kanehisa M (2019). Toward understanding the origin and evolution of cellular organisms. Protein Sci.

[12] Kanehisa M, Furumichi M, Sato Y, Kawashima M, Ishiguro-Watanabe M (2023). KEGG for taxonomy-based analysis of pathways and genomes. Nucleic Acids Res.

[13] Kanehisa M, Goto S (2000). KEGG: kyoto encyclopedia of genes and genomes. Nucleic Acids Res.

[14] Xu J, Ma J, Guan B, Li J, Wang Y, Hu S (2021). LncRNA HCP5 promotes malignant cell behaviors in esophageal squamous cell carcinoma via the PI3K/AKT/mTOR signaling. Cell Cycle.

[15] Nakamura Y, Kanemaru K, Kojima R, Hashimoto Y, Marunouchi T, Oka N, Ogura T, Tanonaka K, Fukami K (2014). Simultaneous loss of phospholipase Cdelta1 and phospholipase Cdelta3 causes cardiomyocyte apoptosis and cardiomyopathy. Cell Death Dis.

[16] Mierke CT (2019). The matrix environmental and cell mechanical properties regulate cell migration and contribute to the invasive phenotype of cancer cells. Rep Prog Phys.

[17] Carneiro BA, El-Deiry WS (2020). Targeting apoptosis in cancer therapy. Nat Rev Clin Oncol.

[18] Dai J, Su Y, Zhong S, Cong L, Liu B, Yang J, Tao Y, He Z, Chen C, Jiang Y (2020). Exosomes: key players in cancer and potential therapeutic strategy. Signal Transduct Target Therapy.

[19] Zanotelli MR, Zhang J, Reinhart-King CA (2021). Mechanoresponsive metabolism in cancer cell migration and metastasis. Cell Metab.

[20] Duff D, Long A (2017). Roles for RACK1 in cancer cell migration and invasion. Cell Signal.

[21] Novikov NM, Zolotaryova SY, Gautreau AM, Denisov EV (2021). Mutational drivers of cancer cell migration and invasion. Br J Cancer.

[22] Yang J, Antin P, Berx G, Blanpain C, Brabletz T, Bronner M, Campbell K, Cano A, Casanova J, Christofori G, Dedhar S, Derynck R, Ford HL, Fuxe J, Garcia de Herreros A, Goodall GJ, Hadjantonakis AK, Huang RYJ, Kalcheim C, Kalluri R, Kang Y, Khew-Goodall Y, Levine H, Liu J, Longmore GD, Mani SA, Massague J, Mayor R, McClay D, Mostov KE, Newgreen DF, Nieto MA, Puisieux A, Runyan R, Savagner P, Stanger B, Stemmler MP, Takahashi Y, Takeichi M, Theveneau E, Thiery JP, Thompson EW, Weinberg RA, Williams ED, Xing J, Zhou BP (2020). Sheng, and E M T I Association. Guidelines and definitions for research on epithelial-mesenchymal transition. Nat Rev Mol Cell Biol.

[23] Huang C, Xu S, Luo Z, Li D, Wang R, Wang T (2022). Epidemiological evidence between Variants in Matrix Metalloproteinases-2, -7, and – 9 and Cancer Risk. Front Oncol.

[24] Michie J, Kearney CJ, Hawkins ED, Silke J, Oliaro J. The Immuno-Modulatory Effects of inhibitor of apoptosis protein antagonists in Cancer Immunotherapy. Cells. 2020;9(1). 10.3390/cells9010207. 10.3390/cells9010207 PMC7017284 31947615

[25] Kashyap D, Garg VK, Goel N (2021). Intrinsic and extrinsic pathways of apoptosis: role in cancer development and prognosis. Adv Protein Chem Struct Biol.

[26] He Y, Sun MM, Zhang GG, Yang J, Chen KS, Xu WW, Li B (2021). Targeting PI3K/Akt signal transduction for cancer therapy. Signal Transduct Target Ther.

[27] Xu J-C, Chen T-Y, Liao L-T, Chen T, Li Q-L, Xu J-X, Hu J-W, Zhou P-H, Zhang Y-Q (2021). NETO2 promotes esophageal cancer progression by inducing proliferation and metastasis via PI3K/AKT and ERK pathway. Int J Biol Sci.

[28] Jin Y, Meng Q, Zhang B, Xie C, Chen X, Tian B, Wang J, Shih T-C, Zhang Y, Cao J, Yang Y, Chen S, Guan X, Chen X, Hong A (2021). Cancer-associated fibroblasts-derived exosomal miR-3656 promotes the development and progression of esophageal squamous cell carcinoma via the ACAP2/PI3K-AKT signaling pathway. Int J Biol Sci.

[29] Luo Q, Du R, Liu W, Huang G, Dong Z, Li X (2022). PI3K/Akt/mTOR signaling pathway: role in esophageal squamous cell Carcinoma, Regulatory Mechanisms and Opportunities for targeted therapy. Front Oncol.

[30] Tanigawa K, Tsukamoto S, Koma Y-I, Kitamura Y, Urakami S, Shimizu M, Fujikawa M, Kodama T, Nishio M, Shigeoka M, Kakeji Y, Yokozaki H (2022). S100A8/A9 Induced by Interaction with Macrophages in esophageal squamous cell Carcinoma promotes the Migration and Invasion of Cancer cells via akt and p38 MAPK pathways. Am J Pathol.

[31] Hu DX, Sun QF, Xu L, Lu HD, Zhang F, Li ZM, Zhang MY (2022). Knockdown of DEAD-box 51 inhibits tumor growth of esophageal squamous cell carcinoma via the PI3K/AKT pathway. World J Gastroenterol.

[32] Wu J, Liu L, Wu F, Qiu L, Luo M, Ke Q, Deng X, Luo Z (2020). Clinical and prognostic implications of P21 (WAF1/CIP1) expression in patients with esophageal Cancer: a systematic review and Meta-analysis. Dis Markers.

[33] Wang L, Han H, Dong L, Wang Z, Qin Y (2021). Function of p21 and its therapeutic effects in esophageal cancer. Oncol Lett.

[34] Yan S, Xu J, Liu B, Ma L, Feng H, Tan H (2021). Long non-coding RNA BCAR4 aggravated proliferation and migration in esophageal squamous cell carcinoma by negatively regulating p53/p21 signaling pathway. Bioengineered.

[35] Chen L, Bi S, Hou J, Zhao Z, Wang C, Xie S (2019). Targeting p21-activated kinase 1 inhibits growth and metastasis via Raf1/MEK1/ERK signaling in esophageal squamous cell carcinoma cells. Cell Commun Signal.

[36] He Z, Chen J, Chen X, Wang H, Tang L, Han C (2021). microRNA-377 acts as a suppressor in esophageal squamous cell carcinoma through CBX3-dependent P53/P21 pathway. J Cell Physiol.

