# Supplementary material for: PLCD3 promotes malignant cell behaviors in esophageal squamous cell carcinoma via the PI3K/AKT/P21 signaling
Source: BMC Cancer. 2023 Sep 29;23:921. doi: 10.1186/s12885-023-11409-w (PMC10542242; doi:10.1186/s12885-023-11409-w)
Supplement: Supplementary file 1 — Supplementary Material 1 [file 12885_2023_11409_MOESM1_ESM.doc]

**Supplementary material: Figure S1. Scoring criteria for immunohistochemical staining.**

1. **staining intensity score**


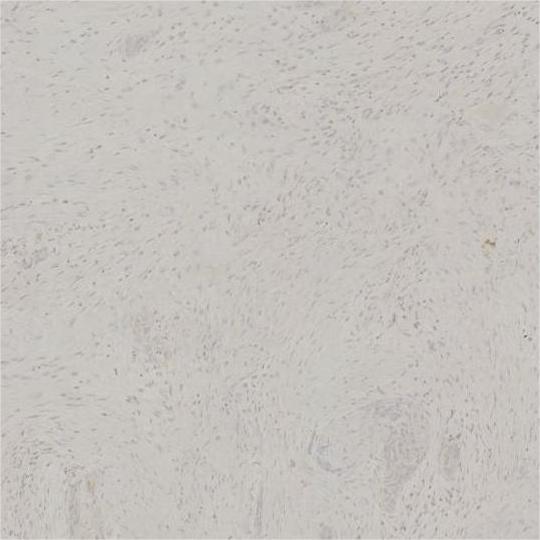

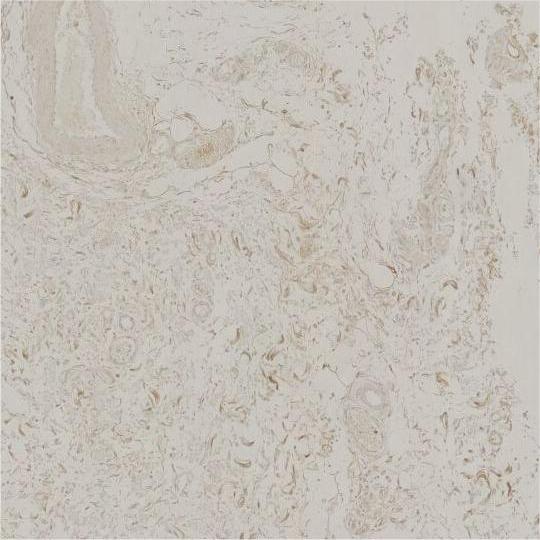

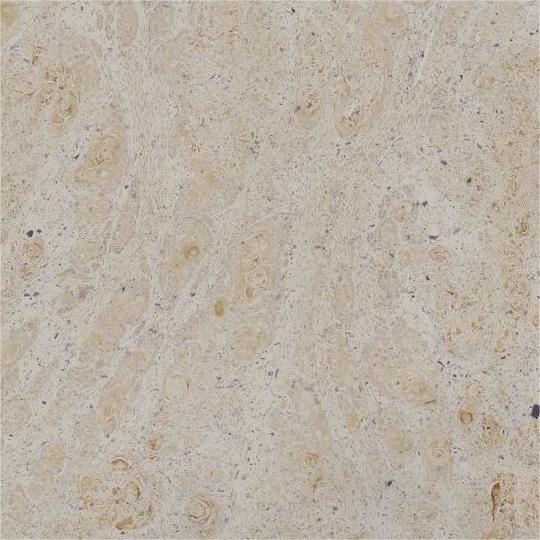

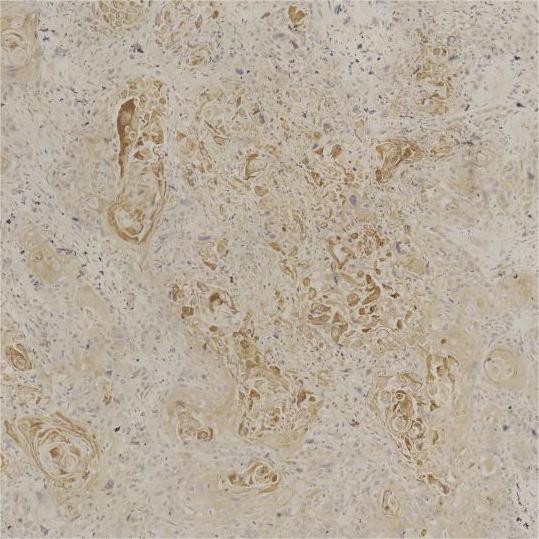


Negative (0) Weak (1) Moderate (2) Strong (3)

1. **positive cell ratio score**

**
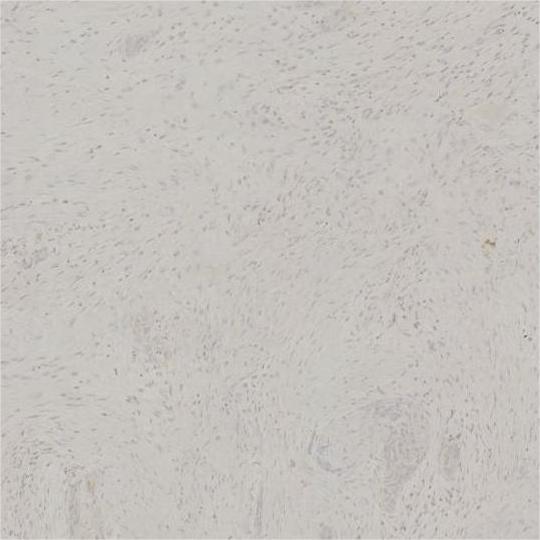

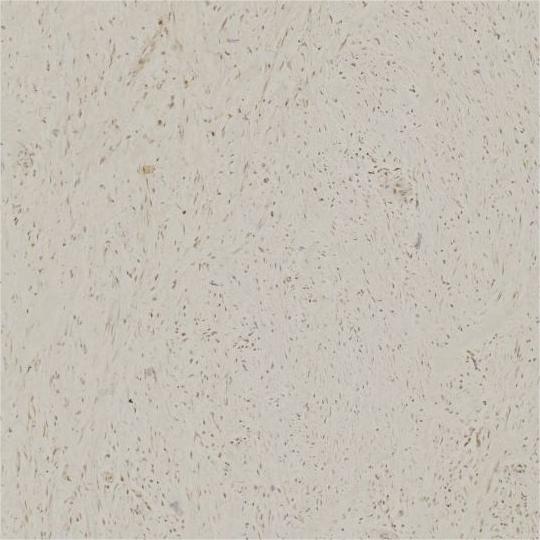

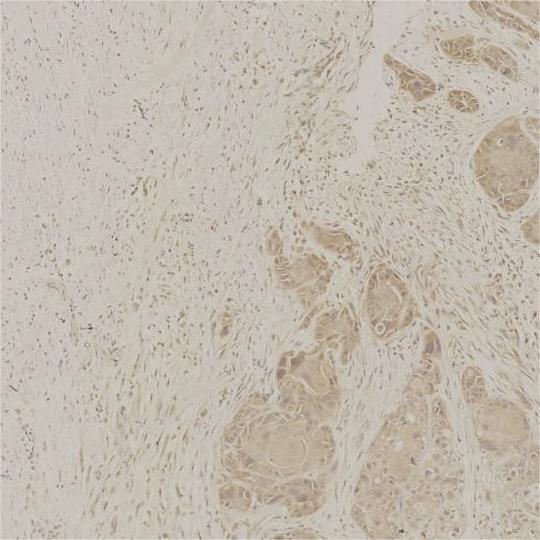
**

0-5%（0） 6-25%（1） 26-50%（2）

**
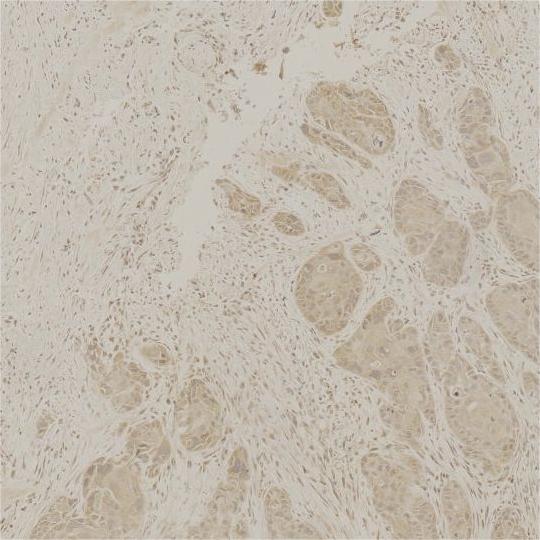
**
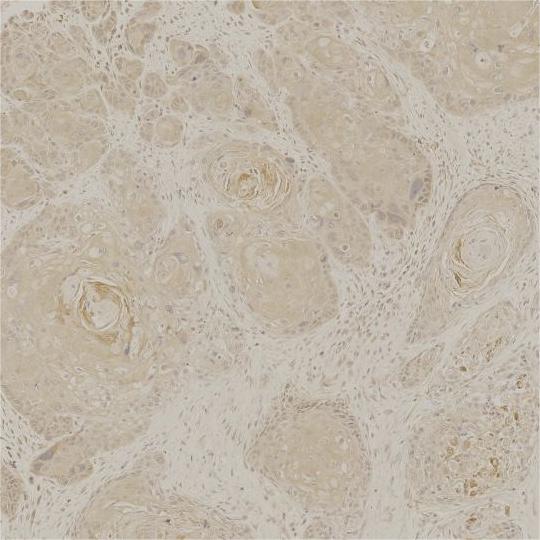


51-75%（3） Over 75%（4）
