# Supplementary material for: PLCD3 promotes malignant cell behaviors in esophageal squamous cell carcinoma via the PI3K/AKT/P21 signaling
Source: BMC Cancer. 2023 Sep 29;23:921. doi: 10.1186/s12885-023-11409-w (PMC10542242; doi:10.1186/s12885-023-11409-w)
Supplement: Supplementary file 2 — Supplementary Material 2 [file 12885_2023_11409_MOESM2_ESM.pdf]

Supplementary material: FigureS2. Original western blot(blots were cut prior to hybridisation with antibodies during blotting)

Figure 1d

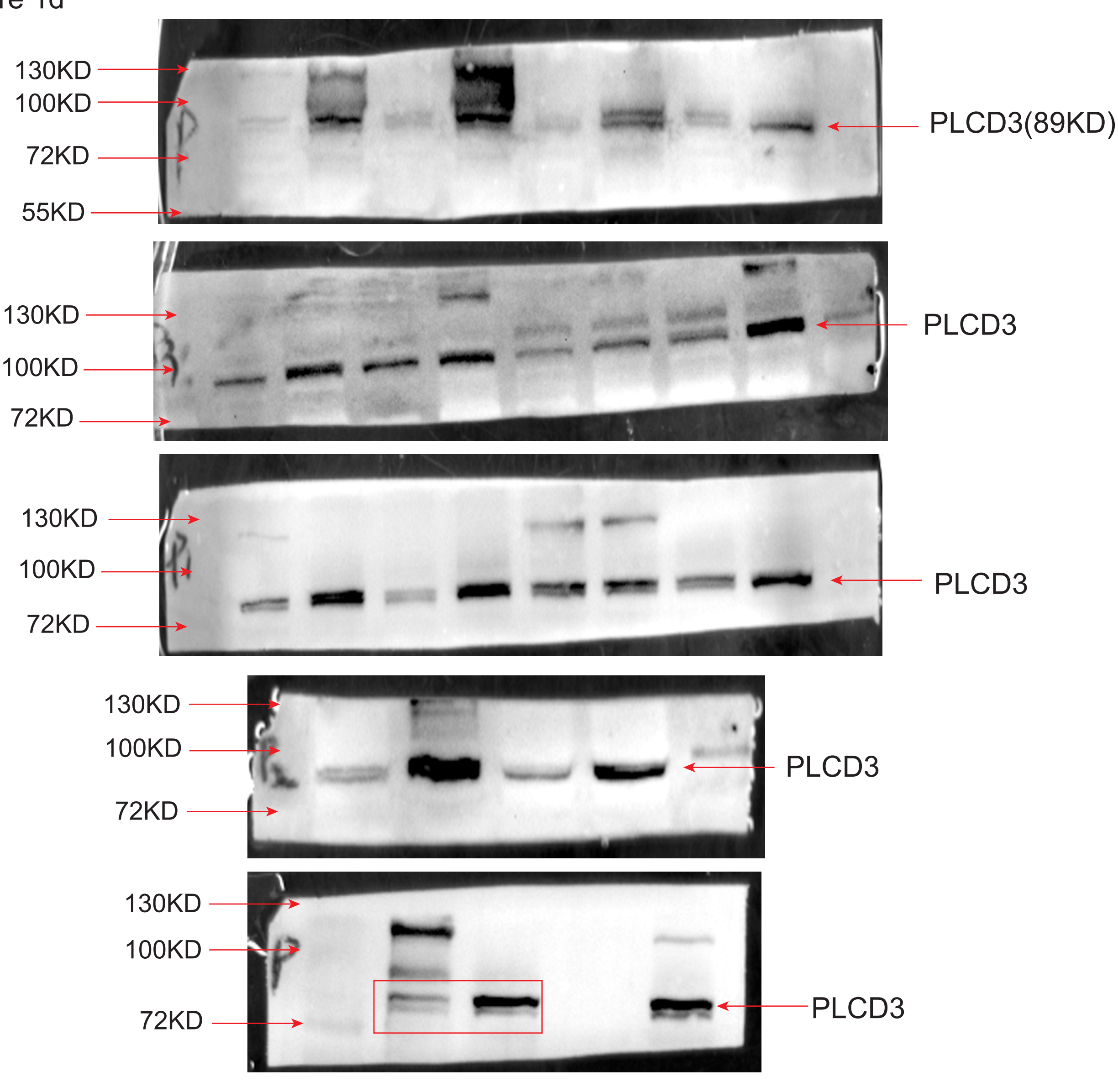

The proteins from left to right come from adjacent non-tumor and tumor tissues of different patients.

Figure 1d

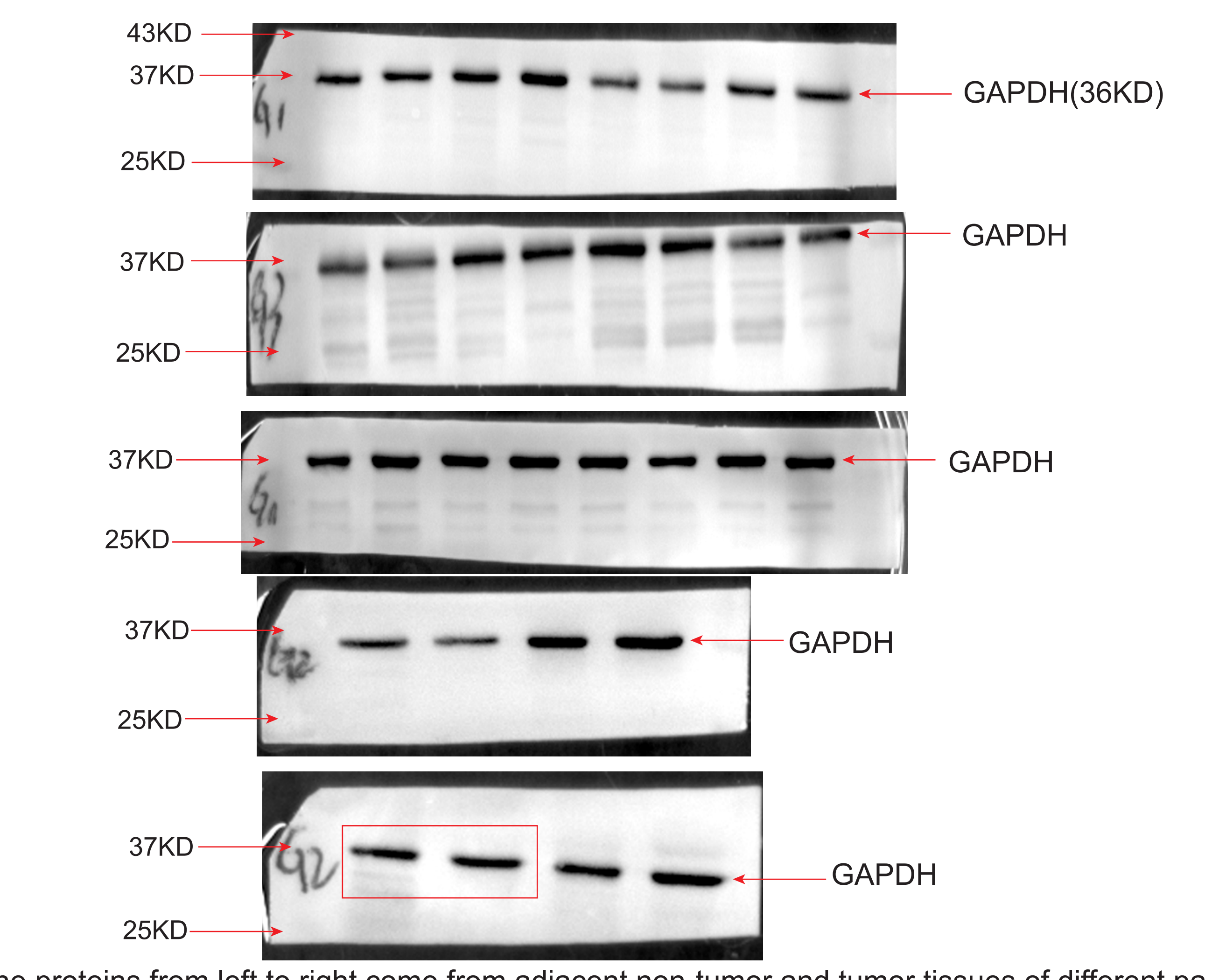

The proteins from left to right come from adjacent non-tumor and tumor tissues of different patients.

Figure 1e

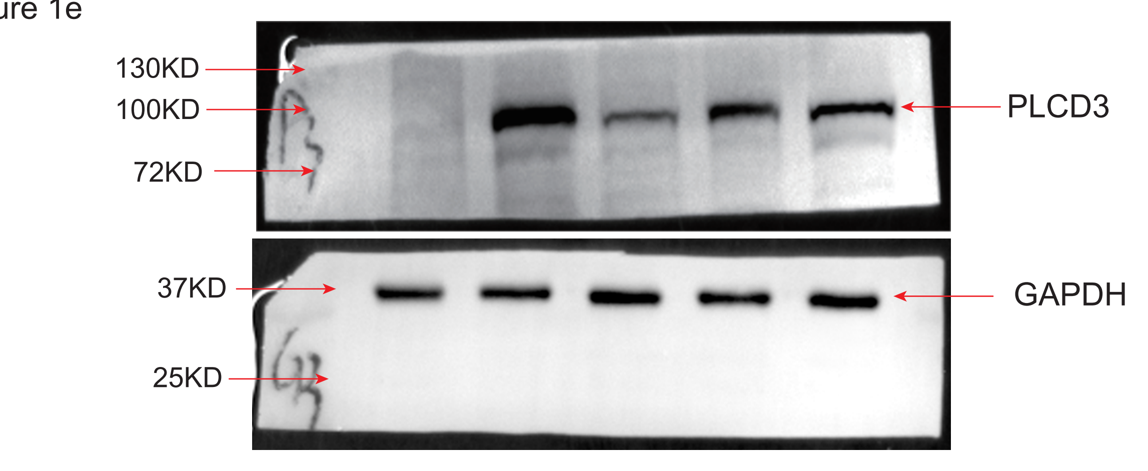

The proteins from left to right come from HEEpiC,ECA109,KYSE150,KYSE410,TE1.

Figure 2b

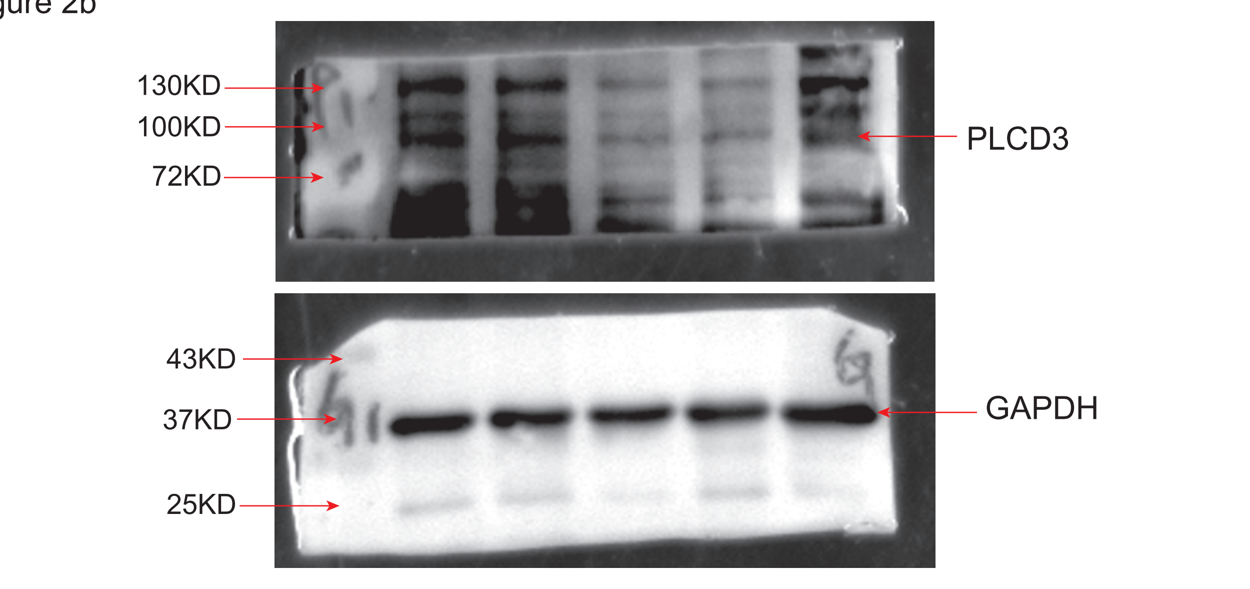

The proteins from left to right come from ECA109 infected with control,siNC,siRNA1,siRNA2 siRNA3.

Figure 2g

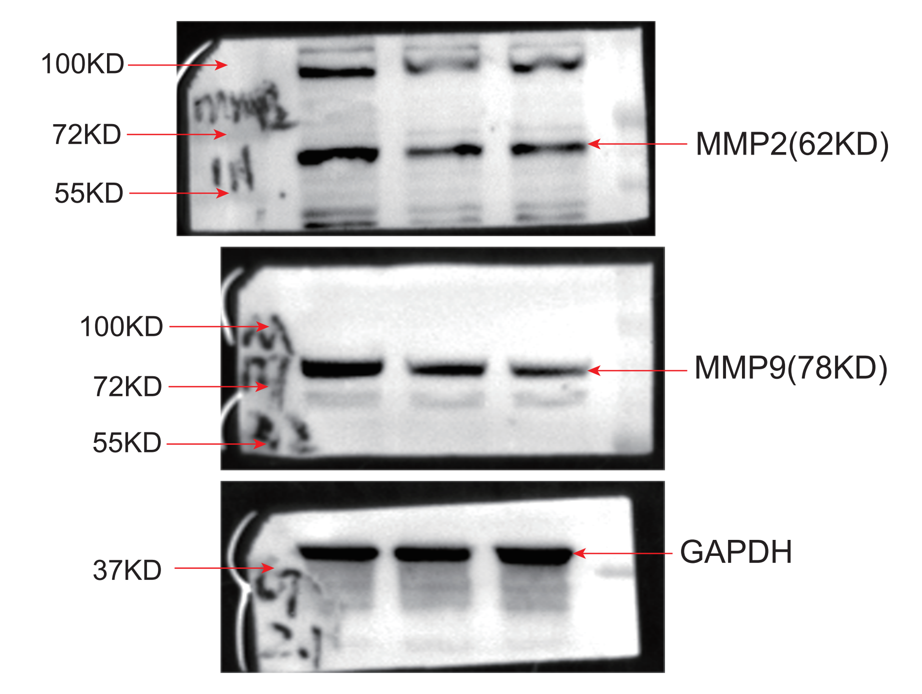

The proteins from left to right come from ECA109 infected with siNC,siRNA2,siRNA3.

Figure 3b

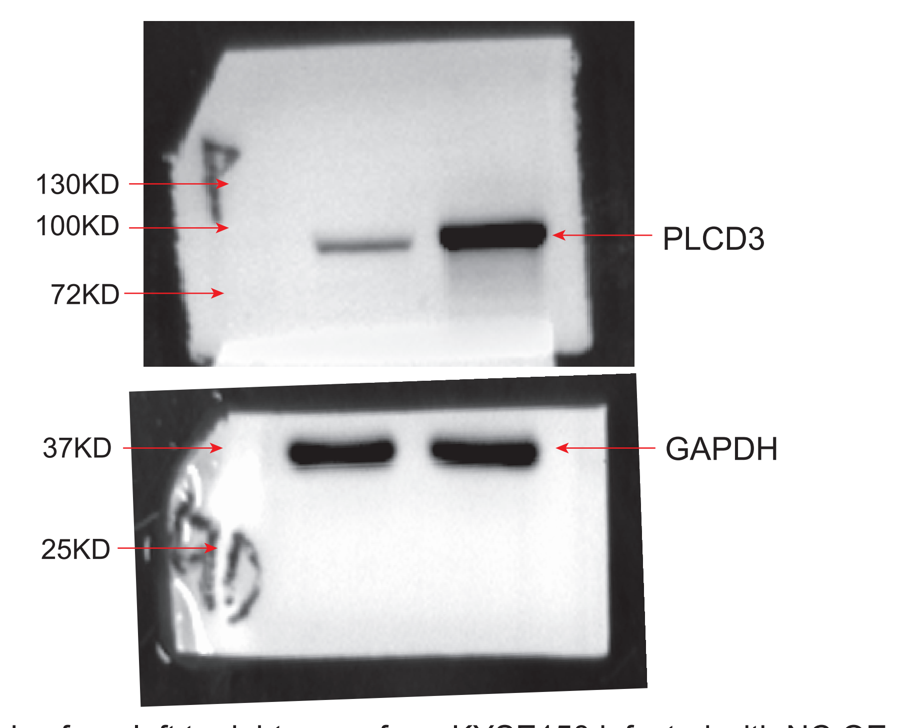

The proteins from left to right come from KYSE150 infected with NC,OE.

Figure 3g

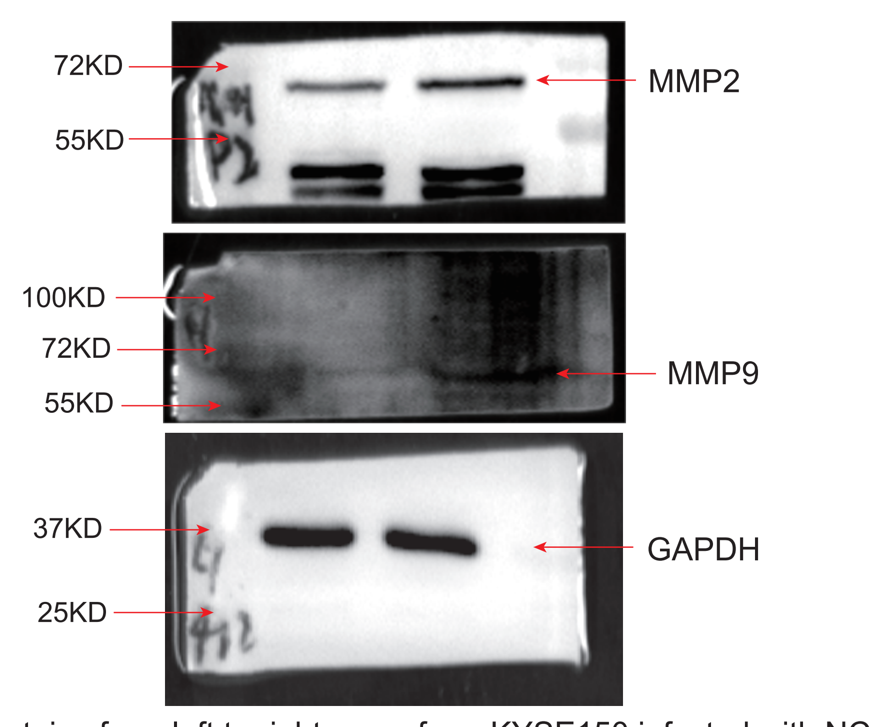

The proteins from left to right come from KYSE150 infected with NC,OE.

Figure 4c

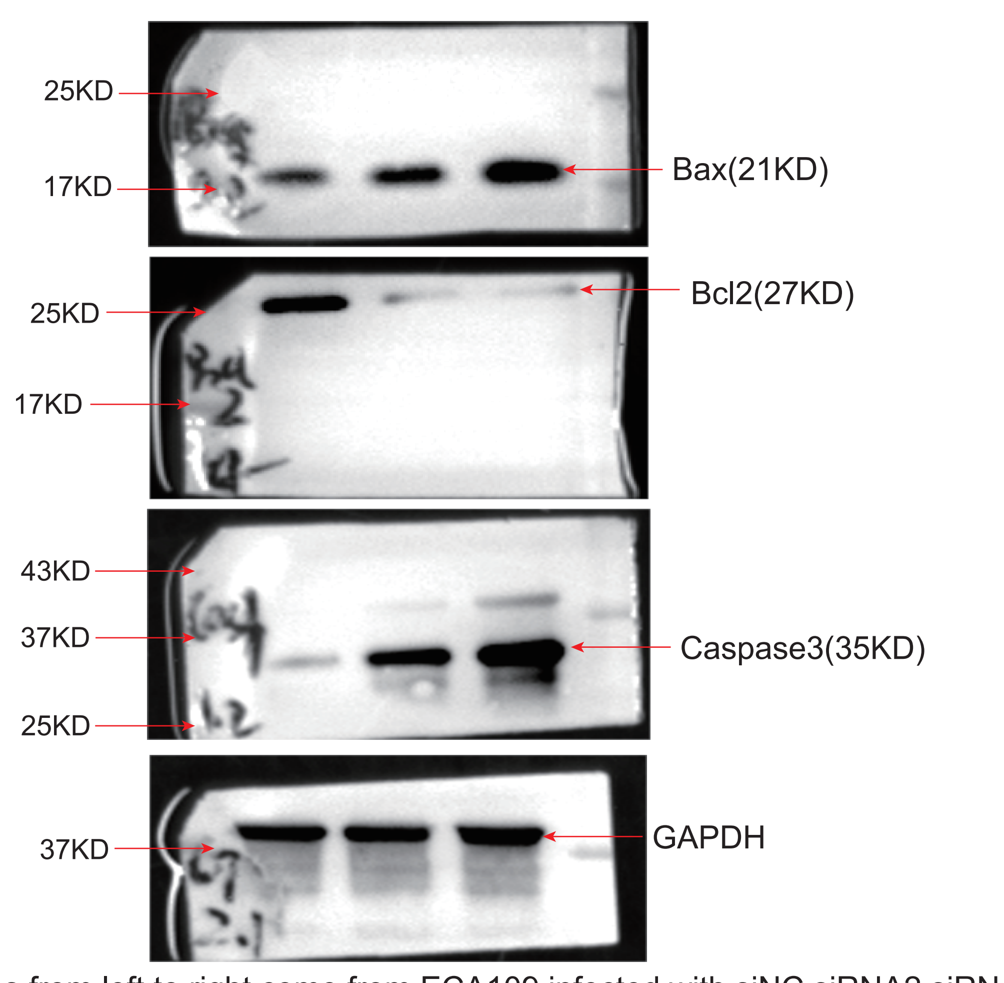

The proteins from left to right come from ECA109 infected with siNC,siRNA2,siRNA3.

Figure 6a

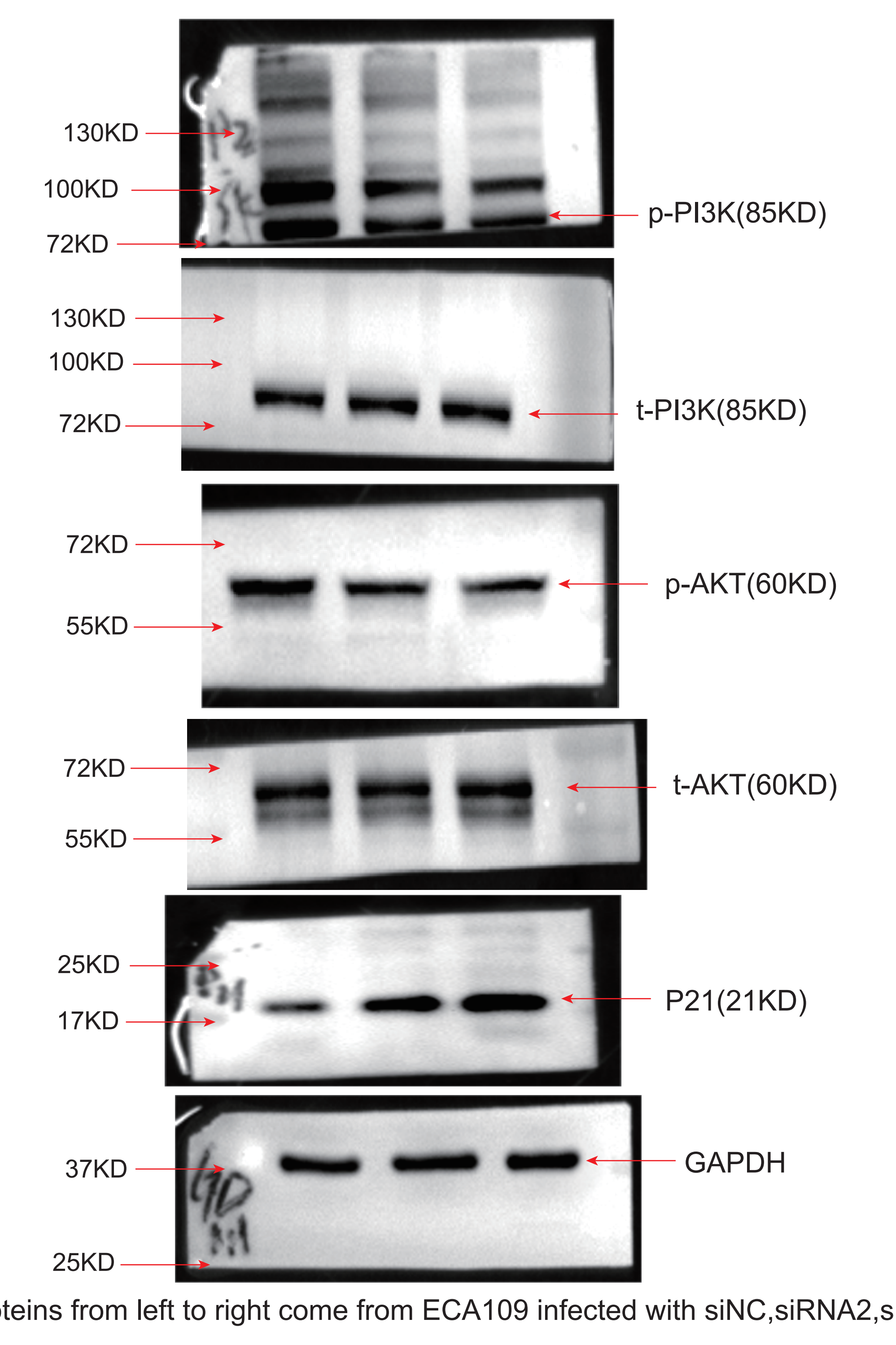

The proteins from left to right come from ECA109 infected with siNC,siRNA2,siRNA3.

Figure 6b

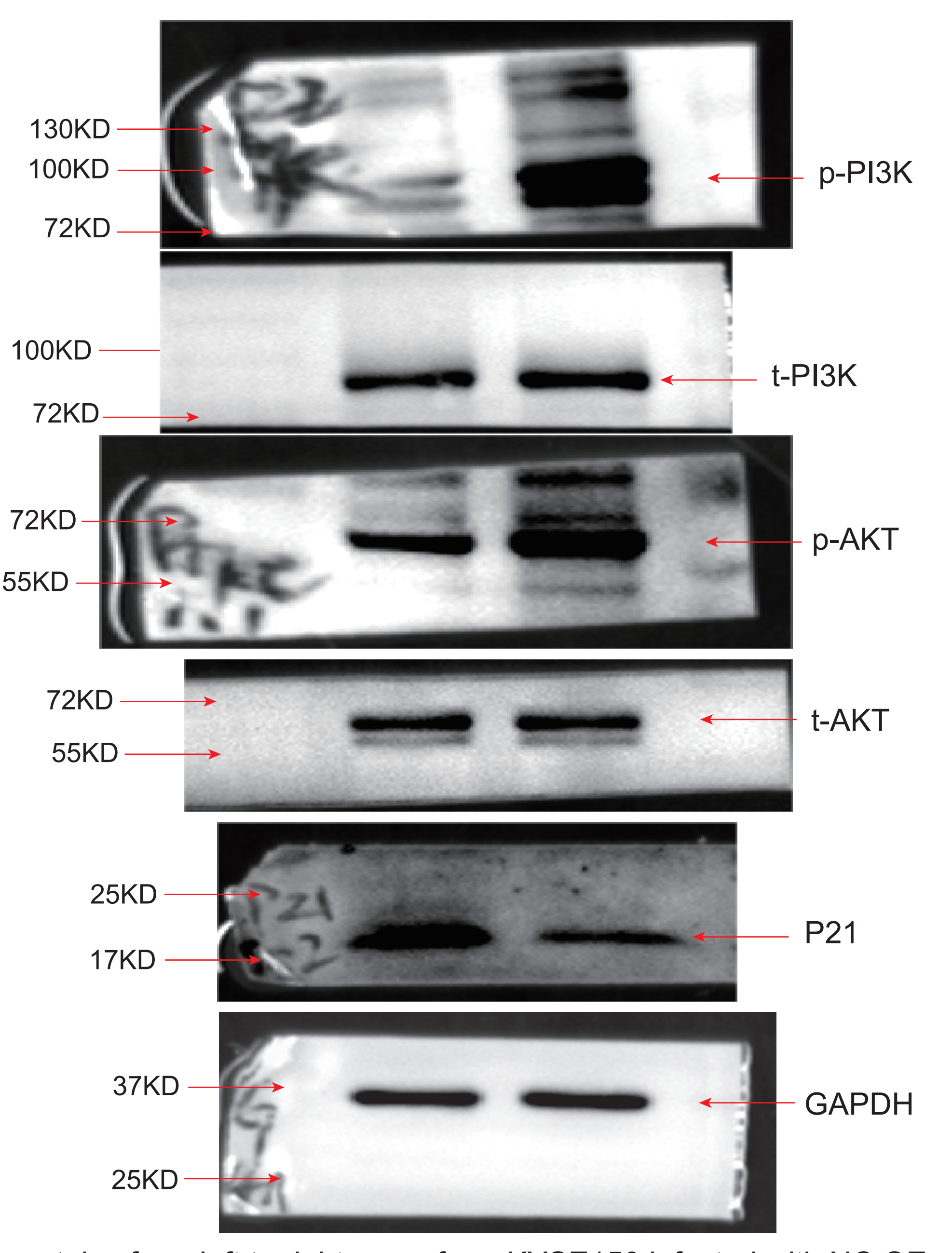

The proteins from left to right come from KYSE150 infected with NC,OE.
